# Tolerance for three commonly administered COVID-19 vaccines by healthcare professionals

**DOI:** 10.3389/fpubh.2022.975781

**Published:** 2022-09-27

**Authors:** Stacy E. F. Melanson, Zhen Zhao, Attila Kumanovics, Tanzy Love, Qing H. Meng, Alan H. B. Wu, Fred Apple, Caitlin R. Ondracek, Karen M. Schulz, Joseph R. Wiencek, David Koch, Robert Christenson, Y. Victoria Zhang

**Affiliations:** ^1^Department of Pathology, Brigham and Women's Hospital, Boston, MA, United States; ^2^Harvard Medical School, Boston, MA, United States; ^3^Department of Laboratory Medicine and Pathology, Weill Cornell Medicine, New York, NY, United States; ^4^Department of Laboratory Medicine and Pathology, Mayo Clinic, Rochester, MN, United States; ^5^Department of Biostatistics and Computational Biology, University of Rochester, Rochester, NY, United States; ^6^Department of Laboratory Medicine, The University of Texas MD Anderson Cancer Center, Houston, TX, United States; ^7^Department of Laboratory Medicine, University of California, San Francisco, San Francisco, CA, United States; ^8^Department of Laboratory Medicine and Pathology, Hennepin Healthcare/Hennepin County Medical Center, Minneapolis, MN, United States; ^9^Hennepin Healthcare Research Institute, Minneapolis, MN, United States; ^10^American Association for Clinical Chemistry, Washington, DC, United States; ^11^Department of Pathology, Microbiology and Immunology, Vanderbilt School of Medicine, Nashville, TN, United States; ^12^Department of Pathology and Laboratory Medicine, Emory University, Atlanta, GA, United States; ^13^Department of Pathology, University of Maryland, Baltimore, MD, United States; ^14^University of Maryland School of Medicine, Baltimore, MD, United States; ^15^Department of Pathology and Laboratory Medicine, University of Rochester Medical Center, Rochester, NY, United States

**Keywords:** COVID-19, SARS-CoV-2, vaccine, Moderna, Pfizer, Johnson and Johnson, side effects

## Abstract

**Importance:**

Most healthcare institutions require employees to be vaccinated against SARS-CoV-2 and many also require at least one booster.

**Objective:**

We determine the impact of vaccine type, demographics, and health conditions on COVID-19 vaccine side effects in healthcare professionals.

**Design:**

A COVID-19 immunity study was performed at the 2021 American Association for Clinical Chemistry Annual Scientific meeting. As part of this study, a REDCap survey with cascading questions was administered from September 9, 2021 to October 20, 2021. General questions included participant demographics, past and present health conditions, smoking, exercise, and medications. COVID-19 specific questions asked about SARS-CoV-2 vaccine status and type, vaccine-associated side effects after each dose including any boosters, previous infection with COVID-19, diagnostic testing performed, and type and severity symptoms of COVID-19.

**Results:**

There were 975 participants (47.1% male, median age of 50 years) who completed the survey. Pfizer was the most commonly administered vaccine (56.4%) followed by Moderna (32.0%) and Johnson & Johnson (7.1%). There were no significant differences in vaccine type received by age, health conditions, smoking, exercise, or type or number of prescription medications. Side effects were reported more frequently after second dose (e.g., Moderna or Pfizer) (54.1%) or single/only dose of Johnson & Johnson (47.8%). Males were significantly more likely to report no side effects (*p* < 0.001), while females were significantly more likely to report injection site reactions (*p* < 0.001), fatigue (*p* < 0.001), headache (*p* < 0.001), muscle pain (*p* < 0.001), chills (*p* = 0.001), fever (*p* = 0.007), and nausea (*p* < 0.001). There was a significant upward trend in participants reporting no side effects with increasing age (*p* < 0.001). There were no significant trends in side effects among different races, ethnicities, health conditions, medications, smoking status or exercise. In multivariate logistic regressions analyses, the second dose of Moderna was associated with a significantly higher risk of side effects than both the second dose of Pfizer and the single dose of Johnson & Johnson.

**Conclusions and relevance:**

Younger people, females, and those receiving the second dose of Moderna had more COVID-19 vaccine side effects that per self-report led to moderate to severe limitations. As reported in other studies, the increase in side effects from Moderna may be explained by higher viral mRNA concentrations but be associated with additional protective immunity.

## Introduction

The availability of vaccines against COVID-19 has changed the course of the pandemic and reduced disease severity (1, 2). In late 2020, several vaccines against the spike protein of SARS-CoV-2 were granted Emergency Use Authorization by the FDA. Both Pfizer (BNT162b2) and Moderna (mRNA-1273), mRNA vaccines, and Johnson & Johnson (J&J), an adenovector, were commonly administered in the United States. Healthcare professionals (HCP) were one of the first populations to be vaccinated leading to many fully vaccinated (e.g., two doses of Moderna or Pfizer or one dose of J&J) HCP by the spring of 2021. Vaccine boosters were available to certain high-risk populations in the summer of 2021 and routinely recommended for adults in the fall of 2021. Following the Centers for Medicare and Medicaid Services mandates, healthcare institutions require employees to be vaccinated against SARS-CoV-2 (3).

Most surveys in the literature assess the attitudes of HCP toward COVID-19 vaccination (4–10). In France and Belgium ~30% of HCP were reluctant to receive vaccinations, primarily because of safety concerns (4). Alley et al. (5) reported that in Australia, women and those without a bachelor's degree were less willing to get vaccinated. In Great Britain, non-white, younger adults with lower education and/or unconfirmed past infection were less likely to get vaccinated (7). Many HCP were concerned about vaccine efficacy, safety, side effects, and speed of vaccine development. Results of these surveys have been utilized to develop targeted education on the benefits of vaccination. Despite targeted education, misinformation remains one common cause of continued vaccine hesitancy (11).

Surveys on vaccine side effects have also been published (12–18). Common side effects to the Pfizer vaccine included soreness, fatigue, myalgia, headache, chills, fever, joint pain, nausea, muscle spasm, sweating, and dizziness (12). Ahsan et al. (13) reported that female HCP and those with known allergies were more likely to report side effects. A study performed in Poland demonstrated a higher rate of side effects with the first dose of AstraZeneca when compared to either dose of Pfizer (18). However, to our knowledge, a survey comparing the side effects of the three most common vaccines administered in the U.S. (Pfizer, Moderna, J&J), and the impact of demographics and health conditions on the risk of side effects, has not been performed. Further, our participants represented a healthy, fully vaccinated, middle-aged, and geographically diverse population. We, therefore, conducted this survey and present our findings in this paper.

## Materials and methods

### Study design and participants

A COVID-19 immunity study (CIS) sponsored by the American Association for Clinical Chemistry (AACC) occurred between September 9, 2021 and October 20, 2021. AACC is a global organization with more than 8,000 members from 105 countries including over 5,000 members from the United States. AACC members were informed via email, social media [including Twitter, Facebook, and the Artery—an AACC online discussion platform with 11,000 active participants (8,000 members and 3,000 non-members able to access only the COVID forum)], or both about enrolling in the study which included a health questionnaire survey and blood draw. The survey portion was designed to gather information from HCP about COVID-19 vaccination and its side effects. The study was approved by the University of Maryland Institutional Review Board. Participants < 18 years old and pregnant women were excluded from study participation. The blood draw portion of the study was performed independently of the survey, had different objectives and will be reported in a separate study.

### CIS survey

The survey was administered through REDCap and contained questions about participant demographics, general health, medications, history of COVID-19, and COVID-19 vaccination status (Supplementary Table 1). The participant's medications were categorized according to the FDA guidelines (Supplementary Table 2) (19).

### Statistical analysis

The survey data was retrieved from the REDCap at the end of the survey period. Basic demographic information and COVID-19 specific questions were analyzed. Of those vaccinated, comparisons were made between participants who received Pfizer, Moderna, and J&J vaccines using Kruskal-Wallis rank sum tests (for continuous data) or chi-squared tests (for categorical data). Due to the heterogeneity and relatively small sample size, participants who received other or unknown vaccine types were not included in the analysis. We focused on typical side effects as described in the CDC and FDA guidelines (20, 21). Health conditions were recorded by detailed disease types; however, they were analyzed at the higher disease category level (e.g., neurological disease) to provide adequate statistical power in each group. Analysis of medication categories was performed if 20 or more participants reported taking a medication in that category.

Logistic regression models were fit to predict the presence of individual side effects after the single dose of J&J and the second dose of Pfizer and Moderna; those doses were completed vaccinations and associated with the highest rate of side effects (referred to as the second/single dose in results). The sensitivity model was adjusted for age, sex, race, ethnicity, self-reporting of overweight, cancer, autoimmune, lung, or other disease, self-reported antidepressant, respiratory tract agent or sex hormone medications, or over-the-counter agents, and previous positive COVID-19 RT-PCR or rapid antigen test. Adjustments for the other health conditions and medication categories, and self-reported healthy, exercise status, and smoking history were considered, but not included in the multivariate model because they were not significant univariate predictors of any side effects. A tertiary model included interactions between vaccine type and both sex and age (in three categories). Odds ratios (OR) for having each side effect were calculated for all comparisons (i.e., Moderna vs. Pfizer, Moderna vs. J&J and Pfizer vs. J&J).

In the logistic regression models, we compared the 932 subjects vaccinated with Pfizer, Moderna, or J&J. For Pfizer and J&J, the risk of any side effects was approximately 50%. Therefore, with at least 80% power, we were able to discover increases in the likelihood of side effects with OR ≥ 1.195 (or decreases ≤ 0.836).

## Results

### Participant demographics, health conditions, and COVID-19 specifics

Of the 1,012 participants who completed the informed consent to answer the health questionnaire survey, 975 completed the survey. Table 1 displays participant demographics, general health, and COVID-19 questions. 47.1% of the participants were male. The median age of the participants was 50 [interquartile range (IQR) 40–59] [male median age 51 (IQR 40–60), female median age 49 (IQR 39–58)]. Most participants were living in the United States (87.4%), Caucasian (77.4%) and non-Hispanic/non-Latino (83.1%). The most common health condition was obesity (17.7%). Nearly all participants (99.1%) responded “yes” when asked if they considered themselves generally healthy (question 19 in Supplementary Table 1). Most had no smoking history (85.9%) and exercised regularly (72.0%). Many participants were taking prescription medications (17.0%), OTC medications/vitamins (19.5%), or both (36.3%) with cardiovascular agents (25.5%) and antidepressants (11.1%) being the most common prescription medications.

**Table 1:** Survey participant demographics, general health conditions, and COVID-19 questions.

Category^*^	**Demographic and general health condition**	**Participants** **(*n* = 975)**
Sex	Male, No. (%)	459 (47.1)
Age	Median (IQR)	50 (40–59)
Country	United States, No. (%)	852 (87.4)
	Canada, No. (%)	21 (2.2)
	Colombia, No. (%)	16 (1.6)
	Mexico, No. (%)	13 (1.3)
	Germany, No. (%)	11 (1.1)
	Other, No. (%)	62 (6.4)
Race	Caucasian, No. (%)	755 (77.4)
	African American/Black, No. (%)	54 (5.5)
	Asian, No. (%)	89 (9.1)
	Native Hawaiian/Pacific Islander, No. (%)	6 (0.6)
	Unknown/Other/Prefer not to say, No. (%)	71 (7.3)
Ethnicity	Hispanic and/or Latino, No. (%)	116 (11.9)
	Non-Hispanic/Non-Latino, No. (%)	810 (83.1)
	Prefer not to reply, No. (%)	49 (5.0)
Health conditions	Overweight or Obesity, No. (%)	173 (17.7)
	Diabetes, No. (%)	58 (5.9)
	Autoimmune disorder, No. (%)	52 (5.3)
	Cancer, No. (%)	50 (5.1)
	Cardiovascular disease, No. (%)	49 (5.0)
	Lung disease, No. (%)	28 (2.9)
	Neurological conditions, No. (%)	11 (1.1)
	Cerebrovascular disease or Stroke, No. (%)	9 (0.9)
	Immunodeficiency, No. (%)	8 (0.8)
	Thalassemia, No. (%)	6 (0.6)
	Liver disease, No. (%)	5 (0.5)
	Solid organ or Blood stem cell transplant, No. (%)	4 (0.4)
	Substance use disorder, No. (%)	4 (0.4)
	Chronic kidney disease, No. (%)	1 (0.1)
	Other, No. (%)	92 (9.4)
	No past or present health conditions, No. (%)	573 (58.8)
Healthy (Per report)	Yes, No. (%)	966 (99.1)
Smoking	Past, No. (%)	121 (12.4)
	Current, No. (%)	16 (1.6)
	No, No. (%)	838 (85.9)
Exercise	Yes, No. (%)	702 (72.0)
	Hours/Week median (IQR)	5 (3–7)
Medications	None, No. (%)	265 (27.2)
	Prescriptions only, No. (%)	166 (17.0)
	OTC/Vitamins only, No. (%)	190 (19.5)
	Both, No. (%)	354 (36.3)
Category^*^		
Number of prescriptions	Median (IQR)	1 (0–2)
Number of OTC/Vitamins	Median (IQR)	1 (0–2)
Types of prescription medications	Cardiovascular agents, No. (%)	249 (25.5)
	Antidepressants, No. (%)	108 (11.1)
	Hormonal agents (thyroid), No. (%)	83 (8.5)
	Respiratory tract agents, No. (%)	66 (6.8)
	Blood glucose regulators, No. (%)	63 (6.3)
	Hormonal agents (sex hormones/modifiers), No. (%)	62 (6.4)
	Gastrointestinal agents, No. (%)	50 (5.1)
	Genitourinary agents, No. (%)	23 (2.4)
Previous COVID infection	Yes (confirmed or suspected), No. (%)	157 (16.1)
	Symptomatic, No. (%)	121 (77.1)
	Severe limitations and/or hospitalization, No. (%)	34 (28.1)
Previous COVID testing (per participant)	Yes, No. (%)	871 (89.3)
	Number, median (IQR)	1 (1, 2)
	Any positive (per participant), No. (%)	98 (11.2)
	Multiple positives, No. (%)	9 (9.1)
Total tests (per test)	All types reported	1,642
	Rapid Antigen Test, No. (%)	496 (30.2)
	RT-PCR, No. (%)	1,032 (62.8)
	Antibody, No. (%)	81 (4.9)
	Unknown, No. (%)	33 (2.0)
	Positive test reported, No. (%)	111 (6.7)
	Rapid Antigen Test, No. (%)	20 (18.0)
	RT-PCR, No. (%)	55 (49.5)
	Antibody, No. (%)	34 (30.6)
	Unknown, No. (%)	2 (1.8)
COVID vaccination	None, No. (%)	9 (0.9)
	Pfizer X2, No. (%)	545 (56.4)
	Moderna X2, No. (%)	309 (32.0)
	J&J X1, No. (%)	69 (7.1)
	AstraZeneca X2, No. (%)	20 (2.1)
	Other, No. (%)	23 (2.4)
COVID booster	Any booster, No. (%)	70 (7.2)
	Pfizer, No. (%)	57 (81.4)
	Moderna, No. (%)	11 (15.7)
	J&J, No. (%)	2 (2.9)
COVID vaccination side effects	Yes (after first dose/single dose), No. (%)	355 (36.7)
	Yes (after second dose), No. (%)	483 (54.1)
	Yes (after booster), No. (%)	36 (51.4)
Post vaccination	Exposure, No. (%)	147/966 (15.2)
	Tested Positive, No. (%)	31/966 (3.2)
	Rapid Antigen Test, No. (%)	5 (16.1)
	RT-PCR, No. (%)	11 (35.5)
	Antibody, No. (%)	2 (6.5)
	Not reported, No. (%)	13 (41.9)

J&J, Johnson & Johnson; OTC, over the counter.

* Refer to Supplementary Table 1 for specific questions asked in each category.

Per self-report, 16.1% of participants had previously suspected or known COVID-19 (48% of those had positive RT-PCR or antigen test). Of the 157 participants reporting previous COVID-19, 121 (77.1%) reported symptoms from the infection, 56 (46.3%) of which led to mild-moderate limitation of activities and 34 (28.1%) of which led to severe limitation of activities including three hospitalizations; one requiring non-invasive ventilation.

The majority of participants (89.3%) were tested for SARS-CoV-2 at some point with a median of one test (IQR = 1–2) and a positivity rate of 11.2% (Table 1). Of all the SARS-CoV-2 testing performed 62.8% was RT-PCR and 30.2% was a rapid antigen test. However, the positive tests were RT-PCR (49.5%), rapid antigen (18.0%), and antibody (30.6%). Of the nine participants who reported multiple positive results, four had a positive PCR result followed by multiple positive serology results, four had positive serology result(s) followed by a positive PCR result, and one had two positive PCR results 13 days apart. Participants reported low rates of post-vaccination exposure (15.2%) with 3.2% testing positive.

### Participant demographics and health conditions by vaccine type

The majority of participants received two doses of the Pfizer vaccine (56.4%), while 32.0% received two doses of Moderna and 7.1% received one dose of J&J (Table 1). The remaining 4.5% received a different vaccine or combination of vaccines, were not fully vaccinated (e.g., only one dose of Moderna) or did not know which vaccine(s) they received (Table 1). At the time of the survey, only 70 (7.2%) participants had received a booster.

Compared to both Pfizer and J&J, Moderna was more frequently administered to males (53.0%; *p* = 0.04) and participants in the United States (94.4%; *p* = 0.007) (Table 2). Participants from Colombia more frequently received Pfizer than Moderna or J&J (*p* = 0.007). Compared to Pfizer or Moderna, J&J was administered to a higher percentage of participants in Mexico (*p* = 0.02). Participants who received J&J were less likely to report that they were healthy (97.7%) as compared to Pfizer (99.1%) and Moderna (100%) (*p* = 0.03). There were no significant differences in vaccine type received by age, health conditions, smoking, exercise, or type or number of prescription medications.

**Table 2:** Participant demographics and health conditions by vaccine type.

Category^*^	**Demographic**	**Pfizer**	**Moderna**	**J&J**	**χ^2^ statistic^†^(df)**	***P*-value^†^**
		**(*n* = 545)**	**(*n* = 309)**	**(*n* = 69)**	
Age	Median (IQR)	50 (41–59)	50 (40–60)	49 (38–57)	1.92 (2)	0.38
Sex	Male, No. (%)	240 (44.0)	164 (**53.0**)	33 (47.8)	**6.46 (2)**	**0.04**
Country	United States (including Puerto Rico), No. (%)	483 (88.6)	293 (**94.8**)	60 (87.0)	**10.01 (2)**	**0.007**
	Canada, No. (%)	11 (2.0)	6 (1.9)	0 (0.0)	1.40 (2)	0.49
	Colombia, No. (%)	14 (**2.6**)	0 (0.0)	0 (0.0)	**9.85 (2)**	**0.007**
	Mexico, No. (%)	7 (1.3)	1 (0.3)	3 (**4.3**)	**7.85 (2)**	**0.02**
	Germany, No. (%)	8 (1.5)	1 (0.3)	0 (0.0)	3.40 (2)	0.18
	Other, No. (%)	22 (4.0)	8 (2.6)	6 (8.7)	5.67 (2)	0.06
Race	Caucasian, No. (%)	431 (79.0)	244 (78.9)	53 (76.8)	8.47 (10)	0.58
	African American/Black, No. (%)	31 (5.6)	17 (5.5)	3 (4.3)		
	Asian, No. (%)	49 (8.9)	30 (9.7)	6 (8.6)		
	Native Hawaiian/Pacific Islander, No. (%)	3 (0.5)	3 (0.9)	0 (0.0)		
	Unknown/Other/Prefer not to say, No. (%)	31 (5.6)	15 (4.8)	7 (10.1)		
Ethnicity	Hispanic and/or Latino, No. (%)	56 (10.3)	31 (10.0)	12 (17.4)	4.60 (4)	0.33
	Non-Hispanic/Non-Latino, No. (%)	469 (86.1)	262 (84.8)	54 (78.3)		
	Prefer not to reply, No. (%)	20 (3.7)	16 (5.2)	3 (4.3)		
Health Conditions	Overweight or Obesity, No. (%)	102 (18.7)	53 (17.1)	11 (15.9)	0.53 (2)	0.76
	Diabetes, No. (%)	37 (6.7)	15 (4.8)	3 (4.3)	1.66 (2)	0.44
	Autoimmune disorder, No. (%)	31 (5.6)	17 (5.5)	4 (5.7)	0.01 (2)	>0.99
	Cancer, No. (%)	28 (5.1)	17 (5.5)	3 (4.3)	0.16 (2)	0.92
	Cardiovascular disease, No. (%)	31 (5.6)	11 (3.5)	2 (2.8)	2.54 (2)	0.28
	Lung disease, No. (%)	16 (2.9)	10 (3.2)	2 (2.8)	0.06 (2)	0.97
	Neurological conditions, No. (%)	5 (0.9)	3 (0.9)	2 (2.8)	2.29 (2)	0.32
	None, No. (%)	312 (57.2)	177 (57.2)	47 (68.1)	3.09 (2)	0.21
Healthy (Per report)	Yes, No. (%)	540 (99.1)	309 (100)	**67 (97.1)**	**6.74 (2)**	**0.03**
Smoking	Past, No. (%)	72 (13.2)	39 (12.6)	4 (5.8)	3.26 (4)	0.52
	Current, No. (%)	9 (1.7)	6 (1.9)	1 (1.4)		
	No, No. (%)	464 (85.1)	264 (85.4)	64 (92.8)		
Exercise	Yes, No. (%)	394 (72.2)	229 (74.1)	53 (76.8)	0.81 (2)	0.66
	Hours/Week median (IQR)	5 (3–7)	5 (3–7)	5 (4–6)	0.61 (2)	0.74
Medications	None, No. (%)	145 (26.6)	81 (26.2)	17 (24.6)	4.92 (6)	0.55
	Prescriptions only, No. (%)	96 (17.6)	53 (17.2)	8 (11.6)		
	OTC/Vitamins only, No. (%)	103 (18.8)	56 (18.1)	20 (28.9)		
	Both, No. (%)	201 (36.9)	119 (38.5)	24 (34.8)		
Number of prescriptions	Median (IQR)	1 (0–2)	1 (0–2)	0 (0–2)	0.77 (2)	0.68
Number of OTC/Vitamins	Median (IQR)	1 (0–2)	1 (0–2)	1 (0–2)	1.04 (2)	0.59
Types of prescription medications	Cardiovascular agents, No. (%)	150 (27.5)	80 (25.8)	13 (18.8)	2.42 (2)	0.30
	Antidepressant, No. (%)	62 (11.3)	31 (10.0)	10 (14.4)	1.19 (2)	0.55
	Hormonal agents (thyroid), No. (%)	48 (8.8)	30 (9.7)	3 (4.3)	2.02 (2)	0.36
	Respiratory tract agents, No. (%)	37 (6.7)	24 (7.7)	4 (5.7)	0.46 (2)	0.79
	Blood glucose regulators, No. (%)	40 (7.3)	17 (5.5)	3 (4.3)	1.66 (2)	0.44
	Hormonal agents (sex hormones/modifiers), No. (%)	35 (6.4)	23 (7.4)	4 (5.7)	0.42 (2)	0.81
	Gastrointestinal agents, No. (%)	32 (5.8)	12 (3.8)	4 (5.7)	1.63 (2)	0.44
	Genitourinary agents, No. (%)	9 (1.6)	10 (3.2)	2 (2.8)	2.35 (2)	0.31

J&J, Johnson & Johnson; OTC, over the counter.

* Refer Supplementary Table 1 for definitions for categories.

† The χ^2^ statistic, df, and p-values refer to a Kruskal-Wallis rank sum test for continuous variables and a chi-square test for categorical variables comparing the three vaccine types. The bold values indicate the statistically significant p-values.

### SARS-CoV-2 vaccine side effects by vaccine type

Participants who received J&J were more likely to have previously had COVID-19 (*p* = 0.006) and experienced a higher likelihood of side effects after the single dose (*p* = 0.003), particularly fatigue (*p* < 0.001), muscle pain (*p* < 0.001), chills (*p* = 0.003), and fever (*p* = 0.006). These side effects led to mild-moderate (*p* < 0.001) or severe (*p* < 0.001) limitation of activities (Table 3). Whereas, Moderna had the highest rate of injection site reactions after the first dose (*p* = 0.001). When compared to the second dose of Pfizer, the second dose of Moderna had a higher rate of side effects (*p* < 0.001) which included injection site reactions (*p* = 0.04), chills (*p* < 0.001), fever (*p* < 0.001), and nausea (*p* = 0.04) which led to mild-moderate limitation of activities (*p* < 0.001). More participants received the Pfizer booster (*p* = 0.004). Although the incidence of most side effects was higher after the Moderna booster than Pfizer, the differences were not significant. There were no significant differences in post-vaccination exposure among the three vaccine types.

**Table 3:** COVID-19 and side effects by vaccine type.

Category^*^	**Demographic**	**Pfizer**	**Moderna**	**J&J**	**χ^2^ statistic^†^(df)**	***P*-value^†^**
		**(*n* = 545)**	**(*n* = 309)**	**(*n* = 69)**		
Previous COVID infection	Yes, No. (%)	79 (14.4)	45 (14.5)	20 (**28.9**)	**10.14 (2)**	**0.006**
	Symptomatic, No. (%)	58 (73.4)	40 (88.8)	15 (75.0)	4.22 (2)	0.12
	Severe limitations and/or hospitalization, No. (%)	19 (32.7)	12 (30.0)	3 (20.0)	0.92 (2)	0.63
COVID vaccination side effects, first dose/single dose	Yes, No. (%)	174 (31.9)	128 (41.4)	33 (**47.8**)	**11.98 (2)**	**0.003**
	Pain/redness/swelling at injection site, No. (%)	111 (20.3)	95 (**30.7**)	12 (17.3)	**13.37 (2)**	**0.001**
	Fatigue, No. (%)	82 (15.0)	73 (23.6)	23 (**33.3**)	**18.77 (2)**	**<0.001**
	Headache, No. (%)	52 (9.5)	36 (11.6)	13 (18.8)	5.67 (2)	0.06
	Muscle pain, No. (%)	43 (7.8)	43 (13.9)	17 (**24.6**)	**20.88 (2)**	**<0.001**
	Chills, No. (%)	27 (4.9)	29 (9.3)	10 (**14.4**)	**11.88 (2)**	**0.003**
	Fever, No. (%)	19 (3.4)	20 (6.4)	8 (**11.5**)	**10.16 (2)**	**0.006**
	Nausea, No. (%)	8 (1.4)	3 (0.9)	3 (4.3)	4.32 (2)	0.11
	Mild-moderate limitation of activities, No. (%)	14 (2.5)	25 (8.0)	10 (**14.4**)	**24.47 (2)**	**<0.001**
	Severe limitation of activities, No. (%)	2 (0.3)	1 (0.3)	3 (**4.3**)	**15.79 (2)**	**<0.001**
	Other, No. (%)	11 (2.0)	5 (1.6)	1 (1.4)	0.23 (2)	0.89
COVID vaccination side effects, second dose	Yes, No. (%)	257 (47.1)	216 (**69.9**)	NA	**40.37 (1)**	**<0.001**
	Pain/redness/swelling at injection site, No. (%)	126 (23.1)	127 (**41.1**)	NA	**4.11 (1)**	**0.04**
	Fatigue, No. (%)	183 (33.5)	166 (53.7)	NA	1.65 (1)	0.20
	Headache, No. (%)	106 (19.4)	90 (29.1)	NA	0.00 (1)	>.99
	Muscle pain, No. (%)	101 (18.5)	104 (33.6)	NA	3.39 (1)	0.07
	Chills, No. (%)	75 (13.7)	108 (**34.9**)	NA	**20.57 (1)**	**<0.001**
	Fever, No. (%)	57 (10.4)	82 (**26.5**)	NA	**13.34 (1)**	**<0.001**
	Nausea, No. (%)	14 (2.5)	24 (**7.7**)	NA	**4.35 (1)**	**0.04**
	Mild-moderate limitation of activities, No. (%)	46 (8.4)	69 (**22.3**)	NA	**11.83 (1)**	**<0.001**
	Severe limitation of activities, No. (%)	12 (2.2)	18 (5.8)	NA	2.07 (1)	0.15
	Other, No. (%)	16 (2.9)	9 (2.9)	NA	0.62 (1)	0.43
COVID booster	Yes, No. (%)	50 (**9.1**)	13 (4.2)	1 (1.4)	**11.01 (2)**	**0.004**
COVID vaccination side effects, booster^1^	Yes, No. (%)	26 (52.0)	9 (69.2)	0 (0.0)	2.46 (2)	0.29
	Pain/redness/swelling at injection site, No. (%)	17 (65.3)	8 (88.8)	NA	0.84 (1)	0.36
	Fatigue, No. (%)	13 (50.0)	5 (55.5)	NA	0.00 (1)	>.99
	Headache, No. (%)	9 (34.6)	4 (44.4)	NA	0.01 (1)	0.90
	Muscle pain, No. (%)	8 (30.7)	3 (33.3)	NA	0.00 (1)	>.99
	Chills, No. (%)	4 (15.3)	4 (44.4)	NA	1.76 (1)	0.18
	Fever, No. (%)	6 (23.0)	2 (22.2)	NA	0.00 (1)	>.99
	Nausea, No. (%)	2 (7.6)	2 (22.2)	NA	0.32 (1)	0.57
	Mild-moderate limitation of activities, No. (%)	6 (23.0)	3 (33.3)	NA	0.02 (1)	0.87
	Severe limitation of activities, No. (%)	0 (0.0)	2 (22.2)	NA	2.69 (1)	0.10
	Other, No. (%)	3 (11.5)	0 (0.0)	NA	0.14 (1)	0.71
Post vaccination exposure1	Yes, No. (%)	87 (15.9)	44 (14.2)	7 (10.1)	1.81 (2)	0.40
	Tested Positive, No. (%)	18 (20.6)	9 (20.4)	1 (14.2)	0.16 (2)	0.92
	Rapid test (Antigen), No. (%)	2 (11.1)	2 (22.2)	1 (100)	5.75 (6)	0.45
	RT-PCR, No. (%)	7 (38.9)	3 (33.3)	0 (0.0)		
	Antibody, No. (%)	1 (5.6)	0 (0.0)	0 (0.0)		
	Not reported, No. (%)	8 (44.4)	4 (44.4)	0 (0.0)		

J&J, Johnson & Johnson.

* Refer Supplementary Table 1 for definitions for categories.

† The χ^2^ statistic, df, and p-values refer to chi-square tests for each categorical variable comparing the three vaccine types. The bold values indicate the statistically significant p-values.

### SARS-CoV-2 vaccine side effects by demographics and health conditions

**Figure 1:**
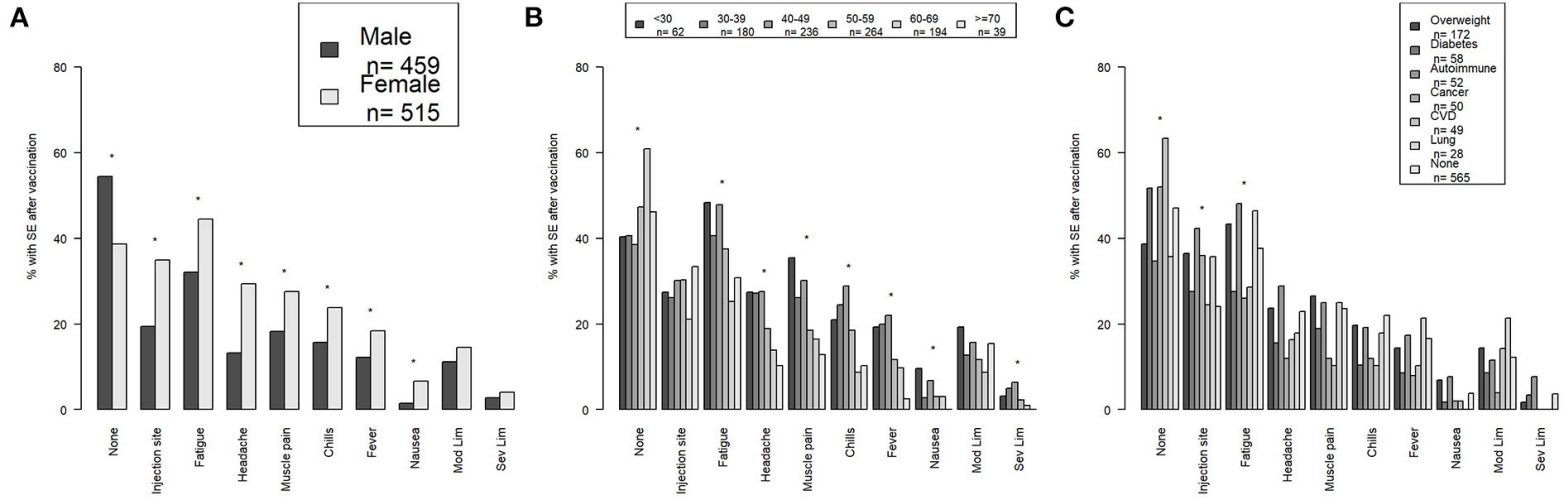
The percentage of no side effects and each specific side effect including mild-moderate and severe limitations by **(A)** sex, **(B)** age group, and **(C)** common health conditions are shown for all vaccinated participants. Side effects after second/single dose are depicted. *Indicates a *p*-value <0.05. SE, side effects; Mod Lim, mild-moderate limitations; Sev Lim, severe limitations; CVD, cardiovascular disease.

Side effects were reported more frequently after second dose (e.g., Moderna or Pfizer) (54.1%) (Table 1) or single dose of J&J (47.8%) (Table 3), therefore in this section we report participant side effects in all participants receiving the second/single dose as a group as well as the side effects per vaccine type. Males were significantly more likely to report no side effects (*p* < 0.001), while females were significantly more likely to report injection site reactions (*p* < 0.001), fatigue (*p* < 0.001), headache (*p* < 0.001), muscle pain (*p* < 0.001), chills (*p* = 0.001), fever (*p* = 0.007), and nausea (*p* < 0.001) (Figure 1A). There were no significant differences in moderate or severe limitations between males and females. Results were similar when Pfizer and Moderna were analyzed separately (Supplementary Figure 1). However, there was no longer a significant difference between males and females for no side effects, muscle pain, or fever for participants who received Moderna. Females who received Pfizer were more likely to report severe limitations (*p* = 0.02).

There was a significant upward trend in participants reporting no side effects with increasing age (*p* < 0.001) (Figure 1B). Conversely, there was a significant downward trend in side effects with increasing age for fatigue (*p* < 0.001), headache (*p* < 0.001), muscle pain (*p* < 0.001), chills (*p* < 0.001), fever (*p* < 0.001), nausea (*p* = 0.03), and severe limitations (*p* = 0.008). There was no significant trend in injection site reactions or moderate limitations among the age groups. Results were similar when Pfizer and Moderna were analyzed separately (Supplementary Figure 2). However, there was no longer a significant trend among the age groups for nausea with either Pfizer or Moderna or for severe limitations for Pfizer. There was a significant downward trend in moderate limitations with increasing age for participants who received Moderna (*p* = 0.009).

African-American/Black participants were less likely to report muscle pain compared to Caucasians and Asians (*p* = 0.04) (Supplementary Figure 3a). There were no other significant differences among the different races. Results were similar when Pfizer and Moderna were analyzed separately (data not shown). However, there was no longer a significant difference between races for muscle pain for participants who received either Moderna or Pfizer. Further, African-American/Black participants who received Pfizer were more likely to report severe limitations (*p* = 0.008).

Non-Hispanic/Non-Latino participants were significantly more likely to report fatigue (*p* = 0.001) and chills (*p* = 0.03) (Supplementary Figure 3b). There were no other significant differences among the different ethnicities. Results were similar when Pfizer and Moderna were analyzed separately (data not shown). However, only fatigue in participants that received Pfizer remained significant (*p* = 0.02).

Participants who reported having diabetes, cancer, and/or cardiovascular disease or no reported health conditions were more likely to have no side effects (*p* = 0.01) (Figure 1C). Participants who reportedly had obesity, autoimmune disease, and cancer and/or lung disease were more likely to report an injection site reaction (*p* < 0.001), while participants who had obesity, autoimmune disease, and/or lung disease or no health conditions were more likely to report fatigue (*p* = 0.003). There were no significant differences in other side effects in participants with the most common health conditions. When Moderna and Pfizer were analyzed separately none of the differences remained significant (Supplementary Figure 4).

Participants taking cardiovascular agents or no medications were significantly less likely to report an injection site reaction (*p* < 0.001), whereas those taking antidepressants, sex hormones/modifiers, and/or respiratory tract agents were more likely to report nausea (*p* < 0.001) (Supplementary Figure 3c). These differences were no longer significant when Pfizer and Moderna were analyzed separately; except for nausea which remained significant for patients taking the medications above who received Moderna (*p* < 0.001) (data not shown). Further, results were similar when participants taking statins were analyzed separately from those taking any cardiovascular agent. There were no significant differences in side effects by smoking status, exercise, exercise duration, and healthy per report (Supplementary Figures 3d–g). Results were similar when Pfizer and Moderna were analyzed separately (data not shown). However, current smokers who received Pfizer were more likely to report chills (*p* < 0.001) and severe limitations (*p* < 0.001).

### Regression models to predict COVID-19 side effects

In a multivariate logistic regression analysis, participants receiving the second dose of Moderna had a significantly higher odds ratio of injection site reactions (OR = 1.22), fatigue (OR = 1.24), headache (OR = 1.13), muscle pain (OR = 1.18), chills (OR = 1.25), fever (OR = 1.19), nausea (OR = 1.06), moderate limitations (OR = 1.15), and severe limitations (OR = 1.03), and lower risk of no side effects (OR = 0.33) when compared to the second dose of Pfizer (Table 4). Similarly, the risk of injection site reactions (OR = 1.28), fatigue (OR = 1.24), headache (OR = 1.15), chills (OR = 1.24), fever (OR = 1.17), and moderate limitations (OR = 1.1) was higher with Moderna than J&J, while the risk of no side effects was lower (OR = 0.33). There were no significant differences in side effects between Pfizer and J&J. Interaction models showed similar findings (Supplementary Tables 3, 4). However, males were more likely to have severe limitations with Moderna as compared to Pfizer.

**Table 4:** Multivariate logistic regression models to predict side effects.

**Side effects**	**Moderna vs. Pfizer**	**J&J vs. Pfizer**	**Moderna vs. J&J**	**Significant covariates**
None	0.33 (0.24, 0.45) *p* < 0.001	0.97 (0.59, 1.69)	0.33 (0.19, 0.58) *p* < 0.001	Age, sex, vitamins/OTC
Injection site	1.22 (1.15, 1.30) *p* < 0.001	0.95 (0.85, 1.06)	1.28 (1.15, 1.44) *p* < 0.001	Sex, vitamins/OTC, previous positive COVID-19
Fatigue	1.24 (1.16, 1.32) *p* < 0.001	1.00 (0.88, 1.12)	1.24 (1.09, 1.41) *p* < 0.001	Age, sex, ethnicity
Headache	1.13 (1.07, 1.20) *p* < 0.001	0.99 (0.89, 1.09)	1.15 (1.03, 1.28) *p* = 0.01	Age, sex, lung disease, previous positive COVID-19
Muscle pain	1.18 (1.11, 1.26) *p* < 0.001	1.07 (0.96, 1.19)	1.11 (0.99, 1.24)	Age, sex
Chills	1.25 (1.18, 1.32) *p* < 0.001	1.01 (0.91, 1.11)	1.24 (1.12, 1.38) *p* < 0.001	Age, sex
Fever	1.19 (1.13, 1.25) *p* < 0.001	1.01 (0.92, 1.11)	1.17 (1.06, 1.29) *p* = 0.001	Age, sex
Nausea	1.06 (1.03, 1.09) *p* < 0.001	1.02 (0.97, 1.07)	1.04 (0.98, 1.10)	Sex, lung disease, antidepressants, sex hormones
Moderate limitation of activities	1.15 (1.10., 1.21) *p* < 0.001	1.05 (0.97, 1.15)	1.10 (1.00, 1.20) *p* = 0.04	Age, previous positive COVID-19
Severe limitation of activities	1.03 (1.00, 1.06) *p* = 0.02	1.02 (0.97, 1.06)	1.02 (0.97, 1.07)	Age, antidepressants

OTC, over the counter; J&J, Johnson & Johnson.

Multivariate model adjusts for age, sex, race, ethnicity, health conditions, medications and previous positive COVID-19 testing. Significant p-values are shown. Values are adjusted odds ratios with 95% confidence intervals and p-values are for the respective odds ratio.

## Discussion

Our survey participants were primarily healthy, middle-aged, non-Hispanic/non-Latino Caucasians from the United States. According to the 2020 census, the United States is 57.8% Caucasian, 12.4% Black, 6% Asian, and 18.7% Hispanic, notably different than our survey participants (22). Approximately 40% of participants reported a health condition, most commonly obesity. Cardiovascular and antidepressant medications represented the majority of prescription medications. However, the health conditions and prescription medications taken by our participants were comparable to the United States population (23). The high rate of antidepressant prescriptions may be explained by the increase in depression, anxiety, and stress in HCP during the pandemic (24–28), though no pre-pandemic data is available for comparison in this particular population.

There was a relatively low number of suspected or confirmed cases of COVID-19 in our participants. However, almost 90% had at least one SARS-CoV-2 test performed. Less than 10% of participants reported multiple positive SARS-CoV-2 results. However, the positive results were either within a short time window or inclusive of a positive PCR test followed by positive antibody results. This suggests the participants were not infected more than once with a different SARS-CoV-2 variant.

A majority (56%) of survey participants received the Pfizer vaccine. This is similar to that seen in the United States (29). Most participants had not received a booster at the time of completing the survey, likely because the booster had not been recommended for healthy adults. Those that received a booster most commonly received Pfizer. Those that received a Moderna booster had higher rates of side effects but the differences were not significant due to the low numbers. Reports from additional participants may provide insights on the impact of COVID-19 boosters and risk of side effects. Post-vaccination exposure was relatively low (15%) suggesting participants may have responded yes only if they met CDC criteria for a close contact (30).

Similar to CDC reports (31–33), injection site reactions, fatigue, headache, and muscle pain were the most common side effects. When comparing the incidence of side effects in the first/single dose group, J&J had a higher rate than Moderna and Pfizer for most side effects including mild to moderate and severe limitations. However, Moderna had a higher rate of injection site reactions after the first dose. The higher incidence of side effects with J&J may be because it is a viral vector, J&J recipients were significantly less healthy per report, and/or J&J recipients had significantly higher rates of previous COVID-19.

Given that the highest rates of side effects were seen after the second/single dose, which is consistent with reports from the CDC and other studies (31–34), we analyzed side effects by demographics and health conditions by grouping second/single dose responses together. Younger and female survey participants had the highest incidence of most side effects regardless of the vaccine administered. Similarly, the CDC (31–33) and Camacho et al. (34) demonstrated higher rates of side effects in younger adults (defined as 18–55 years by CDC and < 50 years by Camacho) and both Ahsan et al. (13) and Camacho et al. (34) reported that females had more side effects. In addition to sex and age, we did not find any other demographics and/or health conditions that had a significant impact on side effects.

In multivariate analysis, the risk of side effects was significantly higher after the second dose of Moderna than after the second dose of Pfizer or a single dose of J&J. These findings were similar to a study by Camacho et al. (34). Although Moderna led to more side effects, Moderna contains a higher concentration of mRNA compared to Pfizer (35) and previous studies suggest that Moderna confers additional protective immunity and leads to fewer breakthrough infections (36–39).

Our study had several limitations. First, our participant population is not representative of the U.S. population, particularly in terms of race, ethnicity, vaccination rates, and co-morbidities. Only 30 states were represented. Second, AACC required vaccination and proof of negative SARS-CoV-2 testing (PCR or antigen) prior to attending the meeting. Therefore, most participants were fully vaccinated. Third, our findings are based on self-reported historical data and are subject to response bias or misinterpretation of questions. Fourth, we were unable to determine, due to the design of the survey, whether patients were positive for SARS-CoV-2 before or after vaccination.

Younger people, females, and those receiving the second dose of Moderna had more COVID-19 vaccine side effects that may have led to moderate to severe limitations. This observation may be explained by higher mRNA concentrations (35). However, as shown in other studies, the increase in side effects may be associated with additional protective immunity and fewer breakthrough infections (36–39).

## Data availability statement

The original contributions presented in the study are included in the article/Supplementary material, further inquiries can be directed to the corresponding author/s.

## Ethics statement

The studies involving human participants were reviewed and approved by University of Maryland Institutional Review Board. The patients/participants provided their written informed consent to participate in this study.

## Author contributions

SM designed the study, reviewed the data, drafted the paper, and critically reviewed the manuscript. ZZ, AK, QM, AW, FA, CO, KS, JW, DK, RC, and YZ designed the study, reviewed the data, and critically reviewed the manuscript. TL performed the data analysis and critically reviewed the manuscript. All authors contributed to the article and approved the submitted version.

## Conflict of interest

Author YZ is a consultant for Thermo Fisher. Author JW is Co-PI on a US and International patent. Specificity enhancing reagents for COVID-19 Antibody Testing. Author ZZ has sponsored research supported by Novartis, Waters, Siemens, Polymedco, Waters, Roche and ET Healthcare and has received consulting/speaker fee from Siemens, Roche and ET Healthcare. The remaining authors declare that the research was conducted in the absence of any commercial or financial relationships that could be construed as a potential conflict of interest.

## Publisher's note

All claims expressed in this article are solely those of the authors and do not necessarily represent those of their affiliated organizations, or those of the publisher, the editors and the reviewers. Any product that may be evaluated in this article, or claim that may be made by its manufacturer, is not guaranteed or endorsed by the publisher.

## References

[1] Watson OJ Barnsley G Toor J Hogan AB Winskill P Ghani AC. Global impact of the first year of COVID-19 vaccination: a mathematical modelling study. Lancet Infect Dis. (2022) 22:1293–302. 10.1016/S1473-3099(22)00320-6 35753318 PMC9225255

[2] Coronavirus Pandemic (COVID-19) - Our World in Data. Available online at: https://ourworldindata.org/coronavirus#explore-the-global-situation (cited August 1, 2022).

[3] CMS. COVID-19 Vaccination Requirements for Health Care Providers and Suppliers. (2021).

[4] Verger P Scronias D Dauby N Adedzi KA Gobert C Bergeat M. Attitudes of healthcare workers towards COVID-19 vaccination: a survey in France and French-speaking parts of Belgium and Canada, 2020. Eurosurveillance. (2021) 26:2002047. 10.2807/1560-7917.ES.2021.26.3.2002047 33478623 PMC7848677

[5] Alley SJ Stanton R Browne M To QG Khalesi S Williams SL. As the pandemic progresses, how does willingness to vaccinate against COVID-19 evolve? Int J Environ Res Public Heal. (2021) 18:797. 10.3390/ijerph18020797 33477825 PMC7832839

[6] Shaw J Stewart T Anderson KB Hanley S Thomas SJ Salmon DA. Assessment of US healthcare personnel attitudes towards coronavirus disease 2019 (COVID-19) vaccination in a large university healthcare system. Clin Infect Dis. (2021) 73:1776–83. 10.1093/cid/ciab054 33491049 PMC7929026

[7] Stead M Jessop C Angus K Bedford H Ussher M Ford A. National survey of attitudes towards and intentions to vaccinate against COVID-19: implications for communications. BMJ Open. (2021) 11:e055085. 10.1136/bmjopen-2021-055085 34711602 PMC8557244

[8] Kuter BJ Browne S Momplaisir FM Feemster KA Shen AK Green-McKenzie J. Perspectives on the receipt of a COVID-19 vaccine: a survey of employees in two large hospitals in Philadelphia. Vaccine. (2021) 39:1693–700. 10.1016/j.vaccine.2021.02.029 33632563 PMC7885691

[9] Ryerson AB Rice CE Hung MC Patel SA Weeks JD Kriss JL. Disparities in COVID-19 vaccination status, intent, and perceived access for noninstitutionalized adults, by disability status — national immunization survey adult COVID module, United States, May 30–June 26, 2021. MMWR Morb Mortal Wkly Rep. (2021) 70:1365–71. 10.15585/mmwr.mm7039a2 34591826 PMC8486390

[10] Mondal P Sinharoy A Su L. Sociodemographic predictors of COVID-19 vaccine acceptance: a nationwide US-based survey study. Public Health. (2021) 198:252–9. 10.1016/j.puhe.2021.07.028 34492505 PMC8318686

[11] Stein RA Ometa O Broker TR. COVID-19: the pseudo-environment and the need for a paradigm change. Germs. (2021) 11:468–77. 10.18683/germs.2021.1283 35096665 PMC8789355

[12] Kadali RAK Janagama R Peruru S Malayala S V. Side effects of BNT162b2 mRNA COVID-19 vaccine: a randomized, cross-sectional study with detailed self-reported symptoms from healthcare workers. Int J Infect Dis. (2021) 106:376–81. 10.1016/j.ijid.2021.04.047 33866000 PMC8049195

[13] Ahsan W Syed NK Alsraeya AA Alhazmi HA Najmi A Bratty M Al. Post-vaccination survey for monitoring the side effects associated with COVID-19 vaccines among healthcare professionals of Jazan province, Saudi Arabia. Saudi Med J. (2021) 42:1341–52. 10.15537/smj.2021.42.12.20210576 34853140 PMC9149761

[14] Djanas D Yusirwan Martini RD Rahmadian Putra H Zanir A. Survey data of COVID-19 vaccine side effects among hospital staff in a national referral hospital in Indonesia. Data Br. (2021) 36:107098. 10.1016/j.dib.2021.107098 33969163 PMC8087582

[15] Perrotta A Biondi-Zoccai G Saade W Miraldi F Morelli A Marullo AG. A snapshot global survey on side effects of cOvid-19 vaccines among healthcare professionals and armed forces with a focus on headache. Panminerva Med. (2021) 63:324–31. 10.23736/S0031-0808.21.04435-9 34738774

[16] Menni C Klaser K May A Polidori L Capdevila J Louca P. Vaccine side-effects and SARS-CoV-2 infection after vaccination in users of the COVID Symptom Study app in the UK: a prospective observational study. Lancet Infect Dis. (2021) 21:939–49. 10.1016/S1473-3099(21)00224-3 33930320 PMC8078878

[17] Kadali RAK Janagama R Peruru S Gajula V Madathala RR Chennaiahgari N. Non-life-threatening adverse effects with COVID-19 mRNA-1273 vaccine: a randomized, cross-sectional study on healthcare workers with detailed self-reported symptoms. J Med Virol. (2021) 93:4420–9. 10.1002/jmv.26996 33822361 PMC8250701

[18] Andrzejczak-Grzadko S Czudy Z Donderska M. Side effects after COVID-19 vaccinations among residents of Poland. Eur Rev Med Pharmacol Sci. (2021) 25:4418–21. 10.26355/eurrev_202106_26153 34227078

[19] *USP Therapeutic Categories Model Guidelines*. FDA. Available online at: https://www.fda.gov/regulatory-information/fdaaa-implementation-chart/usp-therapeutic-categories-model-guidelines (cited August 1, 2022).

[20] Vaccine Adverse Event Reporting System (VAERS). Available online at: https://vaers.hhs.gov/ (cited August 1, 2022).

[21] VAERS. Vaccine Safety. CDC. Available online at: https://www.cdc.gov/vaccinesafety/ensuringsafety/monitoring/vaers/index.html (cited August 1, 2022).

[22] *Census: US Sees Unprecedented Growth Decline in White Population for First Time in History*. Available online at: https://www.usatoday.com/story/news/politics/2021/08/12/how-2020-census-change-how-we-look-america-what-expect/5493043001/

[23] *The 50 Most Commonly Prescribed Drugs in America Their Average Price - DrugReport.com - Reporting on Dangerous Drugs, Defective Medical Devices & Harmful Products*. Available online at: https://www.drugreport.com/50-commonly-prescribed-drugs-in-america/ (cited August 1, 2022).

[24] Hayat K Haq MIU Wang W Khan FU Rehman A ur Rasool MF. Impact of the COVID-19 outbreak on mental health status and associated factors among general population: a cross-sectional study from Pakistan. Psychol Health Med. (2021) 27:54–68. 10.1080/13548506.2021.1884274 33627000

[25] Hayat K Arshed M Fiaz I Afreen U Khan FU Khan TA. Impact of COVID-19 on the mental health of healthcare workers: a cross-sectional study from Pakistan. Front Public Heal. (2021) 9:410. 10.3389/fpubh.2021.603602 33981657 PMC8107369

[26] Huang G Chu H Chen R Liu D Banda KJ O'Brien AP. Prevalence of depression, anxiety, and stress among first responders for medical emergencies during COVID-19 pandemic: a meta-analysis. J Glob Health. (2022) 12:05028. 10.7189/jogh.12.05028 35871411 PMC9309001

[27] Shah AH Becene IA Nguyen KTNH Stuart JJ West MG Berrill JES. A qualitative analysis of psychosocial stressors and health impacts of the COVID-19 pandemic on frontline healthcare personnel in the United States. SSM Qual Res Heal. (2022) 2:100130. 10.1016/j.ssmqr.2022.100130 35873922 PMC9293380

[28] Bucca A Ullrich L Rahman A Smith C Johnson M Allanson-Dundon A. Unmasking the truth of health care workers' well-being during the COVID-19 pandemic. Crit Care Nurse. (2022) 42:e1–7. 10.4037/ccn2022769 35526846

[29] CDC COVID Data Tracker: Vaccinations in the US. Available online at: https://covid.cdc.gov/covid-data-tracker/#vaccinations_vacc-total-admin-rate-total (cited August 1, 2022).

[30] *How To Talk To Your Close Contacts*. CDC. Available online at: https://www.cdc.gov/coronavirus/2019-ncov/daily-life-coping/tell-your-contacts.html#:~:text=For COVID-19%2C,before they were tested (cited August 1, 2022).

[31] Pfizer-BioNTech COVID-19 Vaccine Reactions & Adverse Events. CDC. Available online at: https://www.cdc.gov/vaccines/covid-19/info-by-product/pfizer/reactogenicity.html (cited August 1, 2022).

[32] Moderna COVID-19 Vaccine's Reactions and Adverse Events. CDC. Available online at: https://www.cdc.gov/vaccines/covid-19/info-by-product/moderna/reactogenicity.html (cited August 1, 2022).

[33] The Janssen COVID-19 Vaccine's Local Reactions Systemic Reactions Adverse Events and Serious Adverse Events. CDC. Available online at: https://www.cdc.gov/vaccines/covid-19/info-by-product/janssen/reactogenicity.html (cited August 1, 2022).

[34] Camacho Moll ME Salinas Martínez AM Tovar Cisneros B García Onofre JI Navarrete Floriano G Bermúdez de León M. Extension and severity of self-reported side effects of seven COVID-19 vaccines in Mexican population. Front Public Heal. (2022) 10:387. 10.3389/fpubh.2022.834744 35359754 PMC8964147

[35] Schoenmaker L Witzigmann D Kulkarni JA Verbeke R Kersten G Jiskoot W. mRNA-lipid nanoparticle COVID-19 vaccines: structure and stability. Int J Pharm. (2021) 601:120586. 10.1016/j.ijpharm.2021.120586 33839230 PMC8032477

[36] Lustig Y Gonen T Meltzer L Gilboa M Indenbaum V Cohen C. Superior immunogenicity and effectiveness of the third compared to the second BNT162b2 vaccine dose. Nat Immunol. (2022) 23:940–6. 10.1038/s41590-022-01212-3 35534723

[37] Kaplonek P Cizmeci D Fischinger S Collier AR Suscovich T Linde C. mRNA-1273 and BNT162b2 COVID-19 vaccines elicit antibodies with differences in Fc-mediated effector functions. Sci Transl Med. (2022) 14:2311. 10.1126/scitranslmed.abm2311 35348368 PMC8995030

[38] Puranik A Lenehan PJ Silvert E Niesen MJM Corchado-Garcia J O'Horo JC. Comparison of two highly-effective mRNA vaccines for COVID-19 during periods of Alpha and Delta variant prevalence. medRxiv [Preprint]. (2021). 10.1101/2021.08.06.21261707 34401884 PMC8366801

[39] Tang P Hasan MR Chemaitelly H Yassine HM Benslimane FM Al Khatib HA. BNT162b2 and mRNA-1273 COVID-19 vaccine effectiveness against the SARS-CoV-2 Delta variant in Qatar. Nat Med. (2021) 27:2136–43. 10.1038/s41591-021-01583-4 34728831

