# Supplementary material for: Tolerance for three commonly administered COVID-19 vaccines by healthcare professionals
Source: Front Public Health. 2022 Sep 27;10:975781. doi: 10.3389/fpubh.2022.975781 (PMC9553122; doi:10.3389/fpubh.2022.975781)
Supplement: Supplementary file 1 [file Data_Sheet_1.docx]

**Table of Contents for Supplemental Material**

**eFigure 1 (page 2).** Side effects by gender and vaccine type

**eFigure 2 (page 2).** Side effect by age group and vaccine type

**eFigure 3 (page 3).** Side effects by race, ethnicity, medications, smoking, exercise and healthy per report

**eFigure 4 (page 3).** Side effects by health conditions and vaccine type

**eTable 1 (pages 4-8).** COVID-19 immunity survey questions

**eTable 2 (page 9)**. FDA medication categories

**eTable 3 (page 10).** Side effects and interactions of vaccine type with gender and age (Moderna vs. Pfizer)

**eTable 4 (page 11).** Side effects and interactions of vaccine type with gender and age (Moderna vs. Johnson and Johnson)

**eFigure Legends (page 12).**

**Supplemental Figures**

**
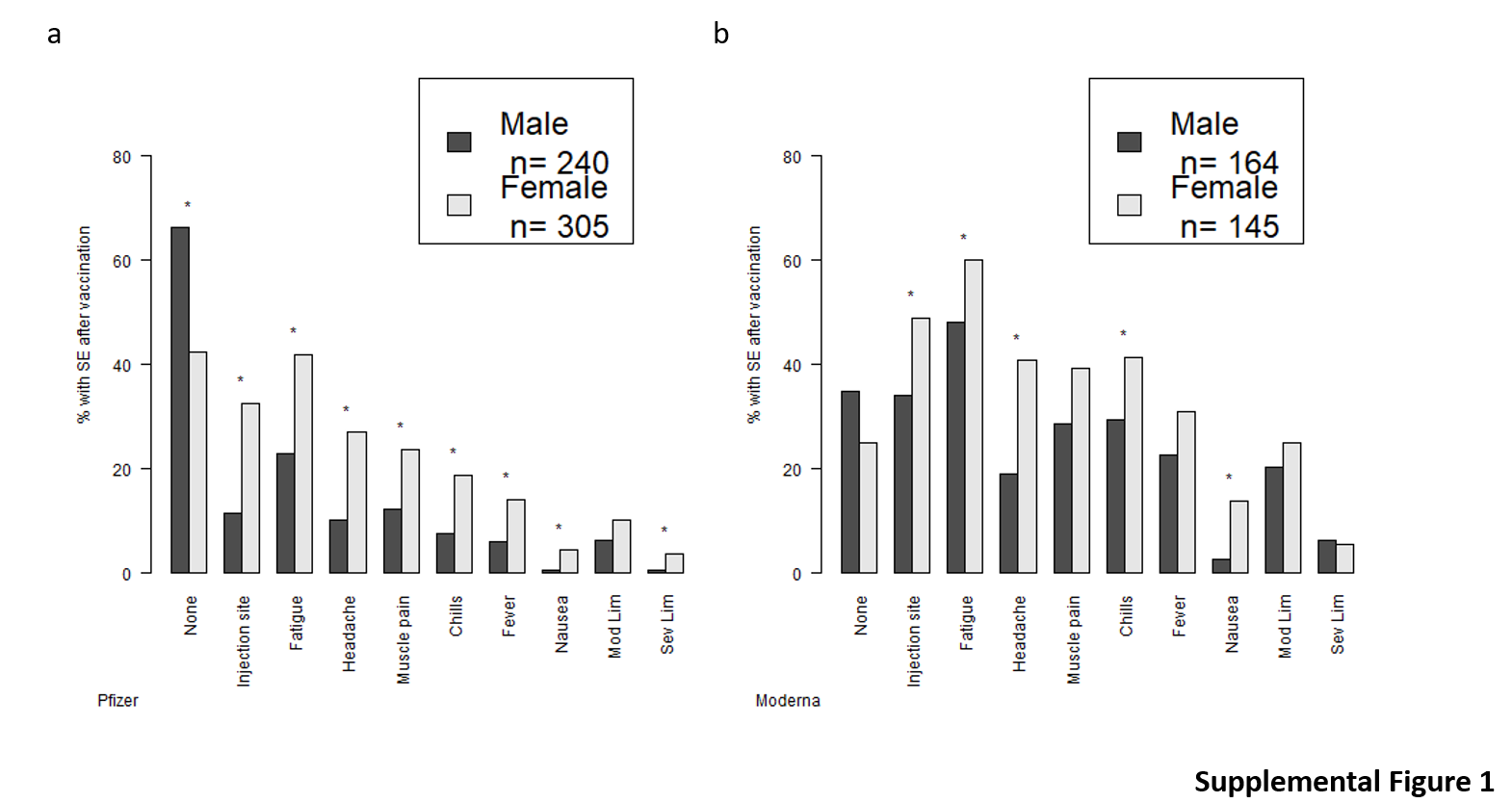
**

**
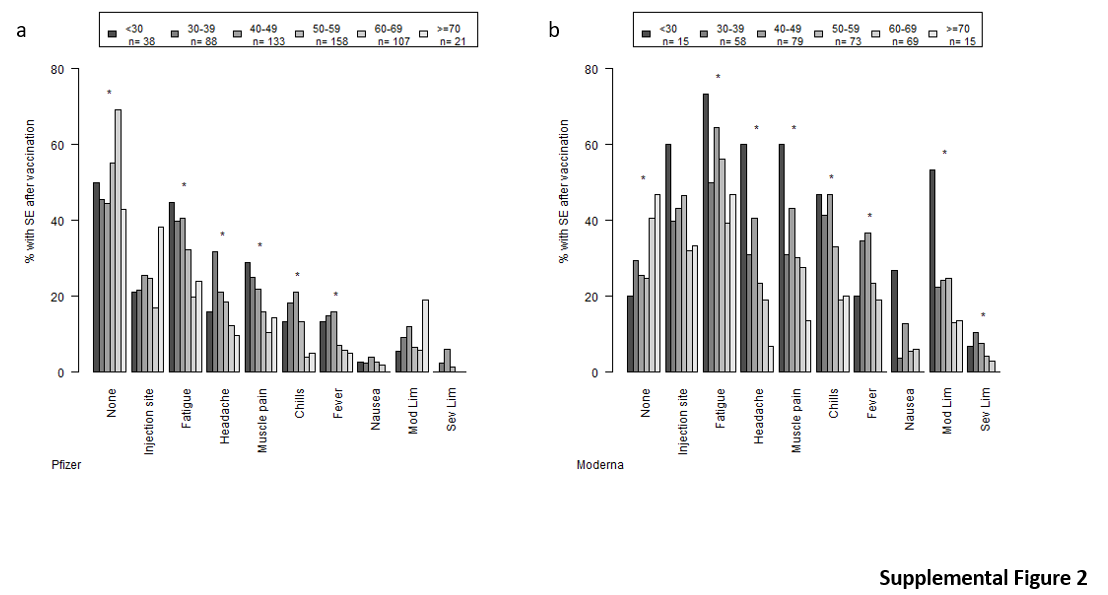
**

**
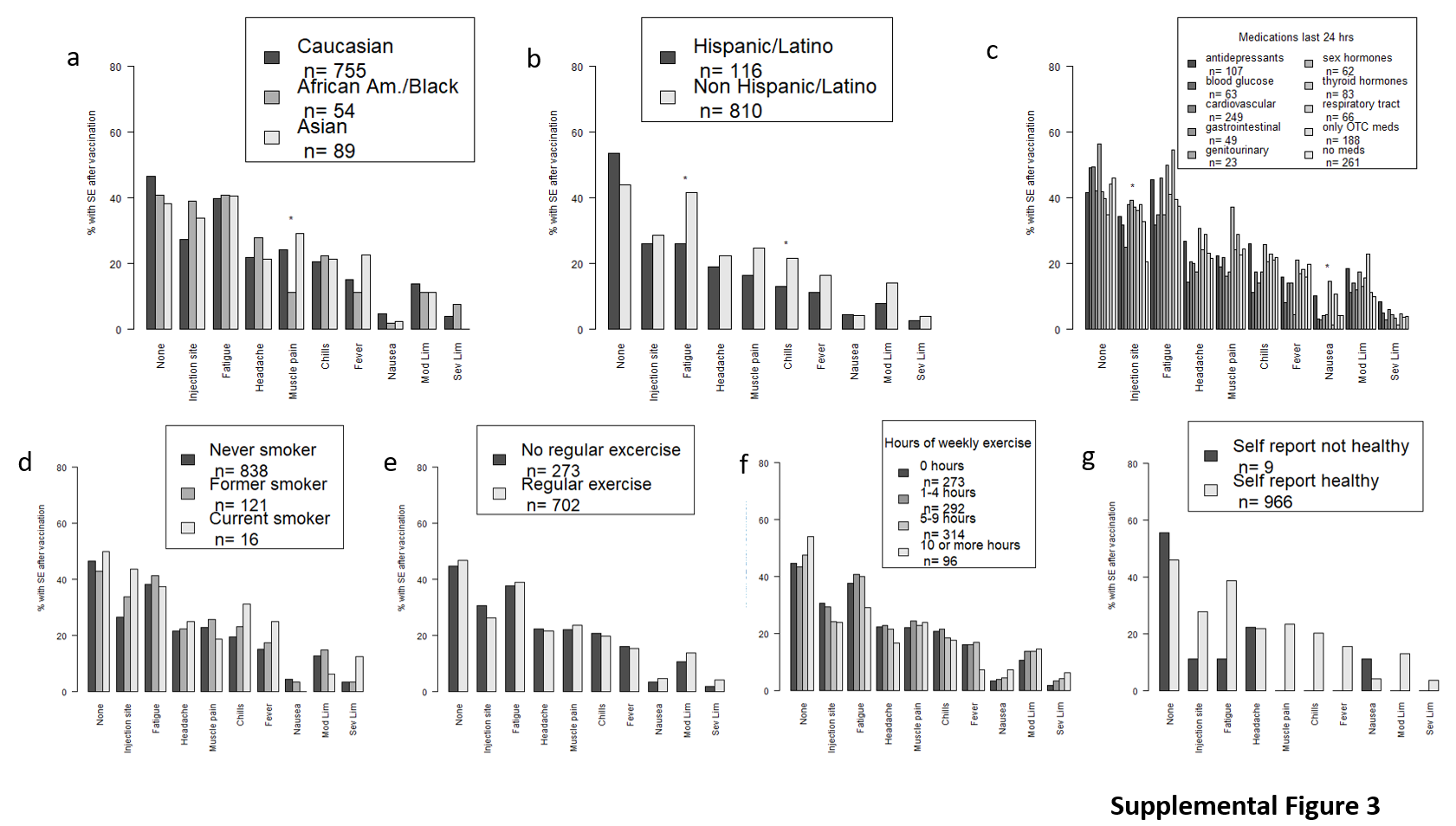
**

**
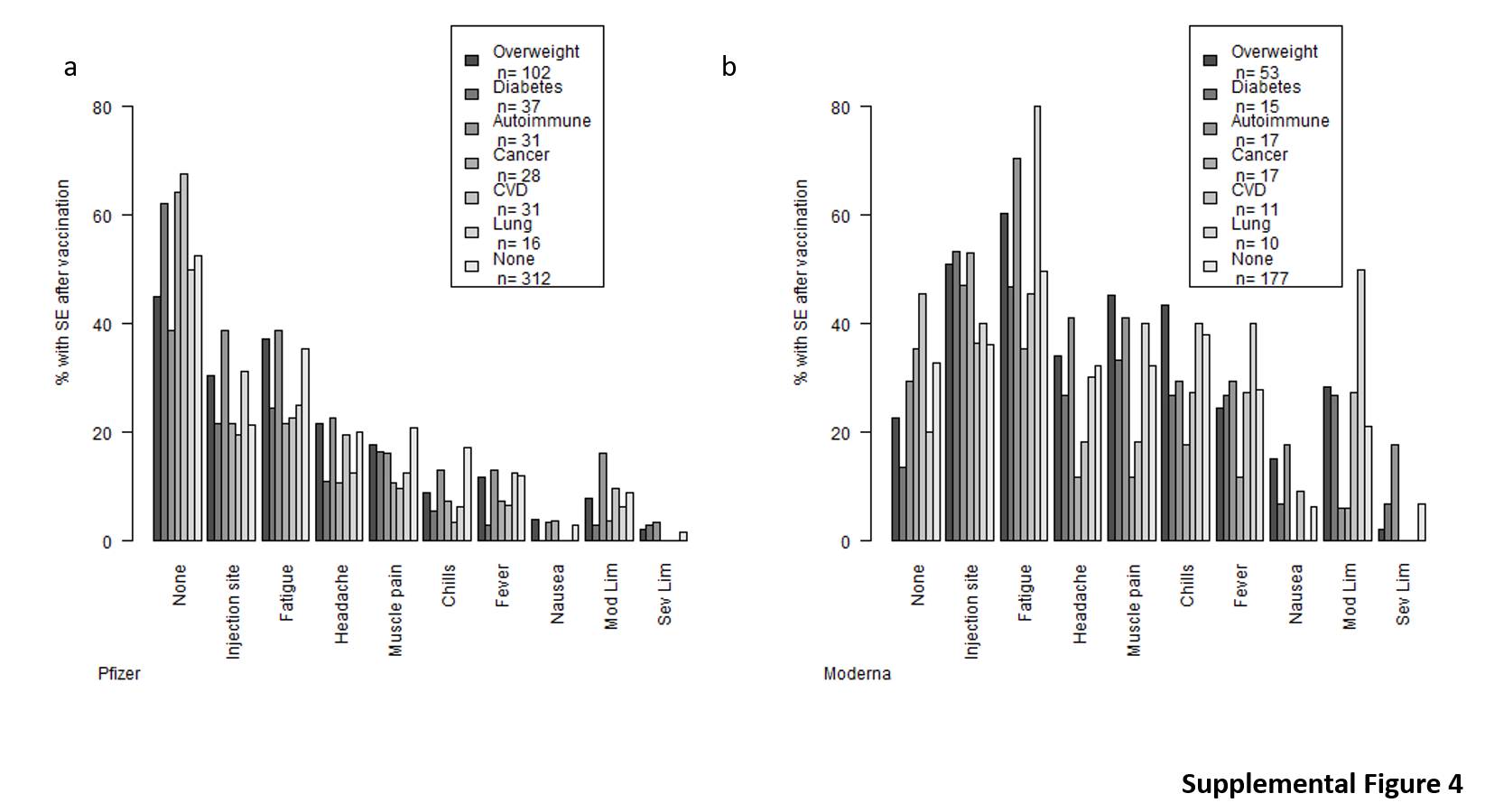
**

**Supplemental Table 1. COVID-19 Immunity Survey Questions**

| **Question #** | **Variable/Field Name and Notes** | **Field Attributes** |
| --- | --- | --- |
| 1 | Age | Text (number, minimum 18) required |
| 2 | Country in which you are currently living | Text required |
| 3 | Postal Code/Zip Code in which you are currently living | Text required |
| 4 | Ethnicity | 1. Hispanic/Latino 2. Non Hispanic/Latino 3. Prefer not to reply |
| 5 | Race | 1. Caucasian 2. African American/Black 3. Asian 4. Native Hawaiian/Pacific Islander 5. American Indian/Alaskan Native 6. Unknown/Other 7. Prefer not to reply |
| 6 | Sex (biological sex recorded at time of birth) | 1. Male 2. Female 3. Prefer not to reply |
| 7 | Pregnancy (only if Sex 2 or 3; currently pregnant) | 1. Yes 2. No |
| 8 | Childbirth (only if Sex 2 or 3; within the past month) | 1. Yes 2. No |
| 9 | Health Conditions (past or present) | 1. Cerebrovascular disease or stroke 2. Neurological condition 3. Lung disease 4. Cardiovascular disease 5. Liver disease 6. Chronic kidney disease 7. Sickle cell disease 8. Thalassemia 9. Diabetes 10. Cancer 11. Immunodeficiency disease 12. Autoimmune disorder 13. Solid organ or blood stem cell transplant 14. Overweight or obesity 15. Substance use disorder 16. Conditions not listed (Text required) 17. No past or present health conditions |
| 10 | Neurological condition (only if health condition 2) | 1. Epilepsy/seizure 2. Amyotropic lateral sclerosis 3. Alzheimers disease 4. Bell’s palsy 5. Guillain-Barre syndrome 6. Multiple Sclerosis 7. Parkinson’s disease |
| **Question #** | **Variable/Field Name and Notes** | 1. **Field Attributes** |
| 11 | Lung disease (only if health condition 3) | 1. Asthma 2. Chronic obstructive pulmonary disease (COPD) 3. Emphysema 4. Other (Text required) |
| 12 | Cardiovascular disease (only if health condition 4) | 1. High blood pressure 2. Cardiomyopathy 3. Coronary artery disease 4. Other (Text required) |
| 13 | Liver disease (only if health condition 5) | 1. Cirrhosis 2. Nonalcoholic fatty liver disease 3. Hepatitis 4. Other (Text required) |
| 14 | Diabetes type (only if health condition 9) | 1. Type 1 2. Type 2 |
| 15 | Cancer (only if health condition 10) | 1. Breast 2. Colon/Rectal 3. Lung 4. Prostate 5. Liver 6. Other (Text required) |
| 16 | Immunodeficiency disease (only if health condition 11) | 1. Primary immunodeficiency 2. Secondary immunodeficiency 3. Other (Text required) |
| 17 | Autoimmune disease (only if health condition 12) | 1. Lupus 2. Rheumatoid arthritis 3. Psoriatic arthritis 4. Crohn’s/Ulcerative colitis 5. Other (Text required) |
| 18 | Type of solid organ/blood stem cell transplant (only if health condition 13) | Text required |
| 19 | Healthy (do you consider yourself generally healthy?) | 1. Yes 2. No |
| 20 | Smoking (Do you have a history of smoking?) | 1. Yes 2. No |
| 21 | Smoker former or current (only if smoking 1) | 1. Former smoker 2. Current smoker |
| 22 | Exercise (do you exercise regularly?) | 1. Yes 2. No |
| 23 | Exercise duration (how many hours do you exercise per week?; only if exercise 1) | Text (number) required |
| 24 | Medications (are you taking any medications?) | 1. Yes 2. No |
| 25 | List medications taken in the past 24-48 hours (only if Medications 1) | Text required |
| 26 | Vitamins and over the counter (OTC) (are you taking any supplements, vitamins or OTC medications?) | 1. Yes 2. No |
| 27 | List supplement(s) or OTC medication(s) taken in the past 24-48 hours (only if Vitamins and OTC 1) | Text required |
| 28 | COVID-19 infection (Have you ever suspected or were confirmed to be infected with COVID-19?) | 1. Yes 2. No |
| **Question #** | **Variable/Field Name and Notes** | 1. **Field Attributes** |
| 29 | COVID-19 symptoms (If you suspected (or were confirmed to have) COVID-19 previously, were you symptomatic (only if COVID-19 infection 1) | 1. Yes 2. No |
| 30 | Did you have any of the following symptoms of COVID-19 (check all that apply) (only if COVID-19 symptoms 1) | 1. Fever 2. Chills 3. Cough 4. Muscle or body aches 5. Fatigue 6. Joint pain 7. Shortness of breath or difficulty breathing 8. Sore throat 9. Runny nose 10. Nasal congestion 11. Sinus infection 12. Nausea or vomiting 13. Diarrhea 14. Headache 15. New loss of taste or smell 16. None of the above |
| 31 | Which best describes your symptomatic state? (only if COVID-19 symptoms 1) | 1. No limitations of activities 2. Mild-moderate limitation of activities 3. Severe limitation of activities 4. Hospitalized; no oxygen therapy 5. Hospitalized; oxygen by mask or nasal prongs 6. Hospitalized; non-invasive ventilation or high-flow oxygen 7. Hospitalized; intubation and mechanical ventilation 8. Hospitalized; ventilation plus additional organ support |
| 32 | COVID-19 testing (Not including this study, have you ever been tested for COVID-19?) | 1. Yes 2. No |
| 33 | Date of previous COVID-19 testing (only if COVID-19 testing 1)* | Text (date_mdy) |
| 34 | COVID-19 test type* | 1. Diagnostic: rapid test (antigen) 2. Diagnostic: RT-PCR 3. Serologic: Antibody test 4. Unknown |
| 35 | COVID-19 test result* | 1. Positive 2. Negative |
| 36 | COVID hospitalization (Due to COVID-19, did you visit the emergency room/hospital/clinic)? | 1. Yes 2. No |
| 37 | Hospitalization date (only if COVID hospitalization 1) | Text (date_mdy) required |
| 38 | Hospitalization length (only if COVID hospitalization 1) | 1. Less than 1 day 2. 1-2 days 3. 3-6 days 4. 1 to 2 weeks 5. 3 weeks to 1 month |
| **Question #** | **Variable/Field Name and Notes** | **Field Attributes** |
| 39 | Hospitalization treatment (only if COVID hospitalization 1) | Text required |
| 40 | COVID-19 vaccination (have you been vaccinated against COVID-19?) | 1. Yes 2. No |
| 41 | COVID-19 vaccination type (only if COVID-19 vaccination 1) | 1. Pfizer-BioNTech 2. Moderna 3. AstraZeneca 4. Johnson & Johnson 5. Unknown 6. Other (Text required) |
| 42 | Date of first vaccination dose (only if COVID-19 vaccination 1) | Text (date_mdy) required |
| 43 | Vaccine side effects, first dose (Did you experience any side effects? (only if first vaccination dose 1) | 1. Yes 2. No |
| 44 | Type of vaccine side effects, first dose (Indicate which side effects (check all that apply) only if vaccine side effects, first dose 1) | 1. Pain/redness/swelling around the injection site 2. Fatigue 3. Headache 4. Muscle pain 5. Chills 6. Fever 7. Nausea 8. Mild-moderate limitation of activities 9. Severe limitation of activities 10. Other (Text required) |
| 45 | Date of second vaccination dose (only if COVID-19 vaccination 1) | Text (date_mdy) required |
| 46 | Vaccine side effects, second dose (Did you experience any side effects? (only if second vaccination dose 1) | 1. Yes 2. No |
| 47 | Type of vaccine side effects, second dose (Indicate which side effects (check all that apply) only if vaccine side effects, second dose 1) | 1. Pain/redness/swelling around the injection site 2. Fatigue 3. Headache 4. Muscle pain 5. Chills 6. Fever 7. Nausea 8. Mild-moderate limitation of activities 9. Severe limitation of activities 10. Other (Text required) |
| 48 | COVID-19 booster (Did you receive a COVID-19 booster shot?) | 1. Yes 2. No |
| 49 | COVID-19 booster type (only if COVID-19 booster 1) | 1. Pfizer-BioNTech 2. Moderna 3. AstraZeneca 4. Johnson & Johnson 5. Unknown 6. Other (Text required) |
| 50 | Booster side effects (Did you experience any side effects? (only if COVID-19 booster 1) | 1. Yes 2. No |
| **Question #** | **Variable/Field Name and Notes** | 1. **Field Attributes** |
| 51 | Type of vaccine side effects, first dose (Indicate which side effects (check all that apply) only if vaccine side effects, first dose 1) | 1. Pain/redness/swelling around the injection site 2. Fatigue 3. Headache 4. Muscle pain 5. Chills 6. Fever 7. Nausea 8. Mild-moderate limitation of activities 9. Severe limitation of activities 10. Other (Text required) |
| 52 | COVID-19 exposure (Do you believe you were exposed to COVID-19 POST vaccination?) | 1. Yes 2. No |
| 53 | COVID-19 post exposure (Did you test positive for COVID-19 POST vaccination; only if COVID-19 exposure 1) | 1. Yes 2. No |
| 54 | Date of POST vaccination testing (only if COVID-19 exposure 1) | Test (date_mdy) |
| 55 | COVID-19 test type (only if COVID-19 exposure 1) | 1. Diagnostic: Rapid test (antigen) 2. Diagnostic: RT-PCR 3. Serologic: Antibody test 4. Unknown |
| 56 | COVID-19 test result (only if COVID-19 exposure 1) | 1. Positive 2. Negative |

^a^The participant is offered the option to answer multiple times to characterize a different occurrence

**Supplemental Table 2. FDA Medication Categories^a^**

| Analgesics |
| --- |
| Anesthetics |
| Antibacterials |
| Anticonvulsants |
| Antidementia agents |
| Antidepressants |
| Antidotes/deterrents/toxicological agents |
| Antiemetics |
| Antifungals |
| Antigout agents |
| Anti-inflammatory agents |
| Antimigraine agents |
| Antimyasthenic agents |
| Antimycobacterials |
| Antineoplastics |
| Antiparasitics |
| Antiparkinson agents |
| Antipsychotics |
| Antispasticity agents |
| Antivirals |
| Anxiolytics |
| Bipolar agents |
| Blood glucose regulators |
| Blood products/modifiers/volume expanders |
| Cardiovascular agents |
| Central nervous system agents |
| Dental/oral agents |
| Dermatological agents |
| Enzyme replacement/modifiers |
| Gastrointestinal agents |
| Genitourinary agents |
| Hormonal agents/stimulant/replacement/modifying (adrenal, pituitary, prostaglandins, sex hormones/modifiers or thyroid) |
| Hormonal agents/suppressant (adrenal, parathyroid, pituitary, sex hormones/modifiers or thyroid) |
| Immunological agents |
| Inflammatory bowel disease agents |
| Metabolic bone disease agents |
| Ophthalmic agents |
| Otic agents |
| Respiratory tract agents |
| Sedative/hypnotics |
| Skeletal muscle relaxants |
| Therapeutic nutrients/minerals/electrolytes |

^a^Some medications may have belonged to more than one category. We selected the most appropriate category based on the participant population. For example, aripiprazole, doxepin, duloxetine, escitalopram, paroxetine, sertraline, and venlafaxine were categorized as antidepressants (as opposed to anxiolytics or antipsychotics). Celecoxib, ibuprofen, meloxicam, and naproxen were categorized as anti-inflammatory (as opposed to analgesics). Lamotrigine was categorized as an anticonvulsant (as opposed to a bipolar agent) and Topiramate was categorized as an antimigraine (as opposed to an anticonvulsant).

**Supplemental Table 3. Side Effects and Interactions of Vaccine type with Gender and Age (Moderna vs. Pfizer)**

| **Side Effects** | **Female** | **Male** | **<45** | **45-55** | **>55** |
| --- | --- | --- | --- | --- | --- |
| None | 0.40 (0.25,0.64)  p<0.001 | 0.26(0.17,0.40)  p<0.001 | 0.35 (0.20, 0.60)  p<0.001 | 0.33 (0.18, 0.60)  p<0.001 | 0.29 (0.17, 0.49)  p<0.001 |
| Injection site | 1.21 (1.11,1.32)  p<0.001 | 1.23 (1.13,1.35)  p<0.001 | 1.28 (1.15, 1.42)  p<0.001 | 1.20 (1.07, 1.34)  p<0.001 | 1.18 (1.06, 1.31)  p<0.001 |
| Fatigue | 1.20 (1.09,1.33)  p<0.001 | 1.27 (1.16,1.40)  p<0.001 | 1.21 (1.08, 1.36)  p<0.001 | 1.27 (1.12, 1.43)  p<0.001 | 1.23 1.10, 1.39)  p<0.001 |
| Headache | 1.16 (1.07,1.26)  p<0.001 | 1.09 (1.01,1.19)  p=0.03 | 1.17 (1.06, 1.29)  p<0.001 | 1.15 (1.04, 1.28)  p=0.01 | 1.06 (0.96, 1.18) |
| Muscle pain | 1.18 (1.09, 1.29)  p<0.001 | 1.18 (1.08, 1.28)  p<0.001 | 1.15 (1.04, 1.28)  p=0.01 | 1.17 (1.05, 1.31)  p<0.001 | 1.21 (1.09, 1.34)  p<0.001 |
| Chills | 1.26 (1.17, 1.37)  p<0.001 | 1.23 (1.14, 1.34)  p<0.001 | 1.33 (1.21, 1.46)  p<0.001 | 1.22 (1.10, 1.35)  p<0.001 | 1.19 (1.08, 1.31)  p<0.001 |
| Fever | 1.19 (1.10, 1.28)  p<0.001 | 1.18 (1.10, 1.27)  p<0.001 | 1.20 (1.10, 1.31)  p<0.001 | 1.21 (1.10, 1.32)  p<0.001 | 1.15 (1.05, 1.26)  p<0.001 |
| Nausea | 1.10 (1.05,1.14)  p<0.001 | 1.02 (0.98, 1.06) | 1.10 (1.04, 1.15)  p<0.001 | 1.02 (0.97, 1.08) | 1.05 (1.00, 1.11)  p=0.04 |
| Moderate  Limitations | 1.16 (1.09, 1.25)  p<0.001 | 1.14 (1.06, 1.22)  p<0.001 | 1.20 (1.11, 1.30)  p<0.001 | 1.15 (1.06, 1.26)  p<0.001 | 1.11 (1.02, 1.20)  p=0.02 |
| Severe  Limitations | 1.01 (0.97, 1.05) | 1.05 (1.01, 1.09)  p=0.01 | 1.06 (1.01, 1.11)  p=0.01 | 1.01 (0.96, 1.06) | 1.03 (0.98 1.07) |

Values are adjusted odds ratios with 95% confidence intervals and p values are for respective odds ratio

**Supplemental Table 4. Side Effects and Interactions of Vaccine type with Gender and Age (Moderna vs. Johnson and Johnson)**

| **Side Effects** | **Female** | **Male** | **<45** | **45-55** | **>55** |
| --- | --- | --- | --- | --- | --- |
| None | 0.23 (0.10, 0.51) p<0.001 | 0.52 (0.24, 1.15)  p<0.001 | 0.53 (0.22, 1.29) | 0.15 (0.04, 0.48)  p<0.001 | 0.33 (0.12, 0.86)  p=0.03 |
| Injection site | 1.37 (1.17, 1.60)  p<0.001 | 1.18 (1.01, 1.39)  p=0.04 | 1.32 (1.12, 1.57)  p<0.001 | 1.28 (1.02, 1.62)  p=0.04 | 1.22 (1.00, 1.49) |
| Fatigue | 1.38 (1.15, 1.64)  p<0.001 | 1.09 (0.91, 1.30) | 1.09 (0.90, 1.31) | 1.44 (1.11, 1.87)  p=0.01 | 1.30 (1.04, 1.62)  p=0.02 |
| Headache | 1.23 (1.06, 1.43)  p=0.01 | 1.06 (0.91, 1.24) | 1.13 (0.96, 1.33) | 1.28 (1.03, 1.60)  p=0.03 | 1.07 (0.89, 1.29) |
| Muscle pain | 1.13 (0.96, 1.32) | 1.09 (0.93, 1.28) | 0.99 (0.84, 1.17) | 1.31 (1.04, 1.65)  p=0.02 | 1.15 (0.95, 1.40) |
| Chills | 1.32 (1.14, 1.53)  p<0.001 | 1.16 (1.00, 1.34) | 1.30 (1.11, 1.52)  p<0.001 | 1.19 (0.96, 1.47) | 1.21 (1.01, 1.45)  p=0.04 |
| Fever | 1.22 (1.06, 1.39)  p<0.001 | 1.12 (0.98, 1.29) | 1.14 (0.99, 1.32) | 1.24 (1.01, 1.51)  p=0.04 | 1.16 (0.98, 1.38) |
| Nausea | 1.12 (1.04, 1.20)  p<0.001 | 0.96 (0.89, 1.04) | 1.01 (0.93, 1.09) | 1.08 (0.97, 1.21) | 1.06 (0.96, 1.16) |
| Moderate  Limitations | 1.06 (0.94, 1.20) | 1.13 (0.99, 1.28) | 1.11 (0.97, 1.27) | 1.14 (0.95, 1.37) | 1.04 (0.89, 1.22) |
| Severe  Limitations | 1.03 (0.96, 1.10) | 1.00 (0.93, 1.07) | 1.03 (0.95, 1.16) | 1.05 (0.95, 1.16) | 0.98 (0.90, 1.07) |

Values are adjusted odds ratios with 95% confidence intervals and p values are for the respective odds ratio

**Supplemental Figure Legends**

**Supplemental Figure 1.** The percentage of no side effects and each specific side effect including mild-moderate and severe limitations by age group is shown for all participants who received a) Pfizer and b) Moderna. Side effects after second/only dose are depicted. * indicates a p value <0.05.

SE = side effects**,** Mod Lim = mild-moderate limitations, Sev Lim = severe limitations

**Supplemental Figure 2.** The percentage of no side effects and each specific side effect including mild-moderate and severe limitations by gender is shown for all participants who received a) Pfizer and b) Moderna. Side effects after second/only dose are depicted. * indicates a p value <0.05.

SE = side effects**,** Mod Lim = mild-moderate limitations, Sev Lim = severe limitations

**Supplemental Figure 3.** The percentage of no side effects and each specific side effect including mild-moderate and severe limitations by a) race, b) ethnicity, c) medications, d) smoking status, e) exercise, f) exercise duration and g) healthy per report is shown for all vaccinated participants. Side effects after second/only dose are depicted. * indicates a p value <0.05.

SE = side effects**,** Mod Lim = mild-moderate limitations, Sev Lim = severe limitations

**Supplemental Figure 4.** The percentage of no side effects and each specific side effect including mild-moderate and severe limitations by the most common health conditions is shown for all participants who received a) Pfizer and b) Moderna. Side effects after second/only dose are depicted. * indicates a p value <0.05.

SE = side effects**,** Mod Lim = mild-moderate limitations, Sev Lim = severe limitations, CVD = cardiovascular disease
